# Rutin-Functionalized Multi-Walled Carbon Nanotubes: Molecular Docking, Physicochemistry and Cytotoxicity in Fibroblasts

**DOI:** 10.3390/toxics9080173

**Published:** 2021-07-22

**Authors:** Conrado M. S. Neto, Felipe C. Lima, Renata P. Morais, Lucas R. M. de Andrade, Renata de Lima, Marco V. Chaud, Matheus M. Pereira, Ricardo L. C. de Albuquerque Júnior, Juliana C. Cardoso, Aleksandra Zielińska, Eliana B. Souto, Álvaro S. Lima, Patrícia Severino

**Affiliations:** 1 Institute of Technology and Research (ITP), University of Tiradentes (UNIT), Av. Murilo Dantas, 300, Aracaju 49010-390, Brazil; conrado.marques@souunit.com.br (C.M.S.N.); felipelima607@gmail.com (F.C.L.); renatamorais04@hotmail.com (R.P.M.); rannier.andrade@outlook.com (L.R.M.d.A.); ricardo_albuquerque@unit.br (R.L.C.d.A.J.); juliana_cordeiro@itp.org.br (J.C.C.); Alvaro_Lima@itp.org.br (Á.S.L.); patricia_severino@itp.org.br (P.S.); 2 School of Bioprocess Engineering and Biotechnology, Sorocaba University, Rodovia Raposo Tavares km 92, Sorocaba 18023-000, São Paulo, Brazil; renata.lima@prof.uniso.br (R.d.L.); marco.chaud@prof.uniso.br (M.V.C.); 3 Department of Chemistry, CICECO—Aveiro Institute of Materials, University of Aveiro, 3810-193 Aveiro, Portugal; matheus.pereira@ua.pt; 4 Institute of Human Genetics, Polish Academy of Sciences Poznan, 60-479 Poznan, Poland; 5 Department of Pharmaceutical Technology, Faculty of Pharmacy, University of Coimbra, Pólo das Ciências da Saúde, Azinhaga de Santa Comba, 3000-548 Coimbra, Portugal; 6 CEB—Centre of Biological Engineering, University of Minho, Campus de Gualtar, 4710-057 Braga, Portugal

**Keywords:** multi-walled carbon nanotubes, rutin, flavonoids, drug delivery, cytotoxicity

## Abstract

Multi-Walled Carbon Nanotubes (MWCNT) have been functionalized with rutin through three steps (i. reaction step; ii. purification step; iii. drying step) and their physicochemical properties investigated with respect to morphological structure, thermal analysis, Fourier Transform Infrared Spectroscopy (FTIR), and cytotoxicity. The molecular docking suggested the rutin-functionalized MWCNT occurred by hydrogen bonds, which was confirmed by FTIR assays, corroborating the results obtained by thermal analyses. A tubular shape, arranged in a three-dimensional structure, could be observed. Mild cytotoxicity observed in 3T3 fibroblasts suggested a dose–effect relationship after exposure. These findings suggest the formation of aggregates of filamentous structures on the cells favoring the cell penetration.

## 1. Introduction

Nanotechnology is being successfully applied in the field of drug delivery, dealing with the combination of a drug with a nanocarrier. The benefits encountered with this approach include the potential to improve the therapeutic effect of the loaded drug, to control its release, absorption and distribution, reduce the risk of systemic toxicity, and also to overcome some limiting physicochemical properties of the drug. Several nanocarriers are described in the literature differing in their size, composition, and administration pathways [1].

Carbon nanotubes (CNTs) have been employed in drug delivery, in particular, to target cancer [2] and neurodegenerative disease [3]. The functionalization of carbon nanotubes through their walls, tips, or encapsulation (open-ended tubes have capillarity) has been seen as a way to explore the potential of CNTs in the pharmaceuticals area. CNTs consist of a carbon sheet weaved into the single or multilamellar cylinder, exhibit excellent electrical conductivity, chemical stability, a large surface area with favorable high encapsulation efficiency, and readily penetrate the cell membranes through barriers by passive diffusion and/or endocytosis [4]. Besides, CNTs present high hydrophobicity attributed to the Van der Waals interactions. The functionalization molecules/drugs or functional groups or molecules, in their walls, overcome the agglomeration and facilitate penetration through cells, viruses, and bacteria. Additionally, this technology is able to create sensors capable of detecting small traces of the target species with high selectivity. Certainly, the development of drug delivery systems by functionalizing CNTs is one of the most promising approaches. The challenge is to find chemically safe, clean, and feasible routes to alter carbon nanotubes that in their natural state have very low chemical reactivity.

Our research group has been focusing on functionalized multi-walled CNTs with secondary metabolites from plants. CNTs have previously been functionalized with naringenin and exhibited high cytotoxicity against human lung alveolar adenocarcinoma (A549) cells compared to free drugs [2]. Besides, CNTs as carriers reduce the cytotoxicity of the healthy cell line (hFB), suggesting their great potential to treat pulmonary adenocarcinomas cancer, but more studies are recommended [5]. Several works describe the use of CNTs as drug delivery systems, attributed to their capacity to improve efficacy and specificity of loaded drugs while reducing the undesirable effects. According to Golubewa et al. (2020) [6], the use of electrical pulses promoted the accumulation of DNA-coated CNTs in the cytoplasm of glioma cells favored by the enhanced permeability and retention (EPR) effect. Other authors suggested the use of laser-mediated ablation of cancer cells marked with biofunctionalized CNTs, which caused necrosis of individual cancer cells and/or induced cell damage through nuclear fragmentation and organelles disintegration [7].

Rutin, also called vitamin P, is a natural phenolic compound (flavonoid) widely found in fruits, seeds, grains, peels, roots, teas, and wines [8]. It is a plant-derived, secondary metabolite that has exhibited a wide range of pharmacological effects, including antioxidant, chemopreventive, anti-inflammatory, anticancer, antiallergic, antiviral [9] and neuroprotective activity [10,11]. Its anticancer activity refers to the potential to modulate oxidative stress, inflammation, autophagy, and apoptosis, leading to the prevention/reduction of their associated toxicity. The generation of free radicals and the pro-oxidant properties of natural agents appear to underlie their direct toxicity to tumor cells. More recently, rutin has been loaded in CNTs [12], maize starch nanoparticles [13], nanocrystals [14] and silver nanoparticles [15]. Rutin silver nanoparticles improved the stability and aqueous solubility of rutin and acted as a nano-anticoagulant with an antithrombotic function showing serum stability, hemocompatibility, and cytocompatibility [15]. Thus, the aim of this work was the functionalization of CNT with rutin, their physicochemical characterization, and the screening of their cytotoxicity profile.

## 2. Materials and Methods

### 2.1. Materials

All materials, including the carbon nanotubes (CAS number 698849), were purchased from Sigma-Aldrich (St. Louis, MO, USA). Rutin was donated by Reis et al. (2014) [16]. Double-distilled water was used after filtration in a Millipore system (home supplied).

### 2.2. Functionalization of CNTs

Functionalization was carried out according to Morais et al., 2020 [2]. The nanotubes were functionalized through three steps (i. Reaction step; ii. Purification step; iii. Drying step). The CNTs were conducted under three different reaction conditions, generating 3 samples, namely, CNT_HP_AA (carbon nanotube treated with ascorbic acid and hydrogen peroxide), CNT_RUT (carbon nanotube and rutin), and CNT_RUT_HP_AA (carbon nanotube treated with ascorbic acid and hydrogen peroxide anchoring the rutin). The sample denominated CNT_RUT_HP_AA was obtained by mixing 0.1 g of rutin, 0.1 g of CNT, and 1.761 g of ascorbic acid in 10 mL of distilled water in the presence of 0.161 mL of hydrogen peroxide. The sample CNT_HP_AA was obtained following the same reaction condition as above, but without the rutin. To generate the sample CNT_RUT, the reaction was carried out with the presence of rutin but without ascorbic acid and hydrogen peroxide. The samples were homogenized with magnetic stirring for 3 h at 25 °C. In the purification stage, the solutions underwent dialysis. Two ml of the solutions were added to each dialysis bag (CNT_RUT_HP_AA, CNT_HP_AA, CNT_RUT); the procedure was carried out in triplicate. In a falcon tube, 20 mL of distilled water was added with the respective dialysis bags accommodated, and within 48 h at a temperature of 25 °C, 8 water changes were performed. For the drying, the samples were submitted to an oven at 110 °C for 24 h.

### 2.3. Molecular Docking

The bidding affinities of CNT_RUT_HP_AA were calculated using the AutoDock Vina 1.1.2 [17,18]. CNT_RUT_HP_AA structure (molecular formula: C_176_H_222_O_60_) was created using Discovery Studio, v20 (Accelrys, San Diego, CA, USA), and applied to Chem3D-MM2 protocol for energy minimization. Auto Dock Tools (ADT) [19] was used to prepare CNT_HP_AA input files by merging non-polar hydrogen atoms, adding partial charges, and atom types. Rutin 3D atomic coordinates were downloaded from ZINC (https://zinc.docking.org/ (accessed on 20 July 2021)). Ligand rigid root was generated using Auto Dock Tools (ADT), setting all possible rotatable bonds defined as active by torsions. The grid center at the center of mass (x-, y-, and z-axes, respectively) of CNT_HP_AA and CNT_RUT_HP_AA was 2.404 Å × −1.711 Å × 0.521 Å. The grid dimension was 30 Å × 30 Å × 30 Å to cover the whole interaction CNT_RUT_HP_AA surface. The binding model that had the lowest binding free energy was searched out from 9 different conformers.

### 2.4. Scanning Electron Microscopy (SEM)

The characterization by scanning electron microscopy (SEM) of the scaffolds was performed using an Electronic Microscope (Ritachi TM 3000, Brazil) with an increase of up to 250×.

### 2.5. Thermal Analysis

Thermogravimetry (TG/DTA) and Differential Scanning Calorimetry (DSC) were performed using the Shimadzu DTG-60H and DSC-60 equipment, with a heating rate of 10 °C/min between 10 and 500 °C, in atmosphere nitrogen. For the analysis, 1.5 mg of samples were used (CNT_HP_AA, CNT_RUT, and CNT_RUT_HP_AA). The DSC was carried out to study phase transitions and exothermic/endothermic decompositions occurring in the investigated samples.

### 2.6. Fourier Transform Infrared Spectroscopy (FTIR)

The infrared spectrum was used in the range of 400–4000 cm^−1^, with KBr tablets, and in the Nicolet FTIR thermal spectrophotometer at room temperature. The samples were prepared by gently mixing 10 mg of each sample with 300 mg of KBr powder, pressed into discs with a force of 17 kN for 5 min, using a manual tablet press.

### 2.7. Cytotoxicity Assay

The cell line 3T3 (embryonic fibroblast) was used to evaluate via the agar disk diffusion methodology [20]. The visual analysis was performed and the clear halo of dead cells around the samples characterized the presence of a toxic material. The cells were plated in 60 mm Petri dishes, at a concentration of 1.5 × 10^5^ cells/mL using DMEM High culture medium with 10% fetal bovine serum, they were allowed to grow for 48 h at 37 °C in a greenhouse 5% CO_2_. Then, the liquid medium was discarded, and the solid “overlay” medium (2× concentrated Eagle medium, containing 1.8% agar and 0.01% neutral red as a vital dye) was added to the cell mat. After solidification, the test material was deposited in the center of the plate and incubated for 24 h at 37 °C in an environment of 5% CO_2_. The readings of the inoculated plates were performed macroscopically, where cytotoxicity was verified by a clear halo around the toxic material corresponding to the dead cells, and microscopically through the morphological changes of the cells surrounding the sample.

## 3. Results and Discussion

**Figure 1:**
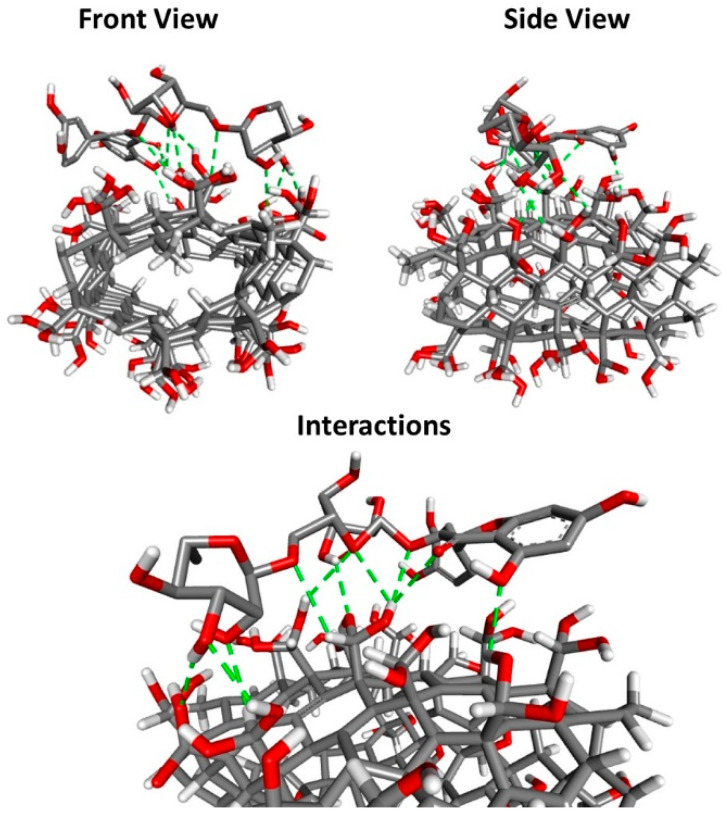
The docking pose with the lowest absolute value of affinity (kcal/mol) for CNT_RUT_HP_AA (Front view, side view, and interactions): Carbon (grey), Oxygen (red), Hydrogen (White) and H-bond (green).

Carbon nanotubes can be covalently functionalized through concentrated acids that are capable of breaking the interactions between carbon atoms. This acid treatment alters the surface of the CNT, enabling the incorporation of charged groups [21]. Mostly used functionalization processes include oxidation through the immersion of the CNT in sulfuric acid and/or nitric acid, where the generation of bonds of carboxylic groups, hydroxyl groups, and sulfonic acids on the surface of the CNT occurs. The reaction conditions (ultrasound, temperature, and function) and oxidizing agents used for the surface functionalization of the CNT are determining factors for the formation of the necessary group bonds [21,22]. The outer surface of the CNT, when modified, can directly interact with biological organic molecules (DNA, RNA, and proteins), with other important exogenous molecules (drugs, polymers, and toxic agents) and even microorganisms and viruses [23,24,25,26,27]. In our work, we used acid ascorbic as an oxidant and H_2_O_2_ as an oxidizing agent. We used 0.1 g CNT, 18 mg ascorbic acid, 10 mL water, and 2 μL H_2_O_2_. Molecular docking was carried out to explore the binding mode between rutin and CNT. The docking pose with the lowest absolute value of affinity (kcal/mol) for CNT_RUT_HP_AA is depicted in Figure 1. The results of docking affinities, type of interaction, and geometry distance (Å) are shown in Table 1.

According to the obtained results, it is suggested that the hydroxyl and oxygen groups ligand between rutin and CNT contributes to the stabilization of the flavonoid. The binding energy of the generated was −8.5 kcal/mol present and the smallest geometric distance (1.96 Å) kcal/mol) from rutin to CNT_HP_AA.

**Figure 2:**
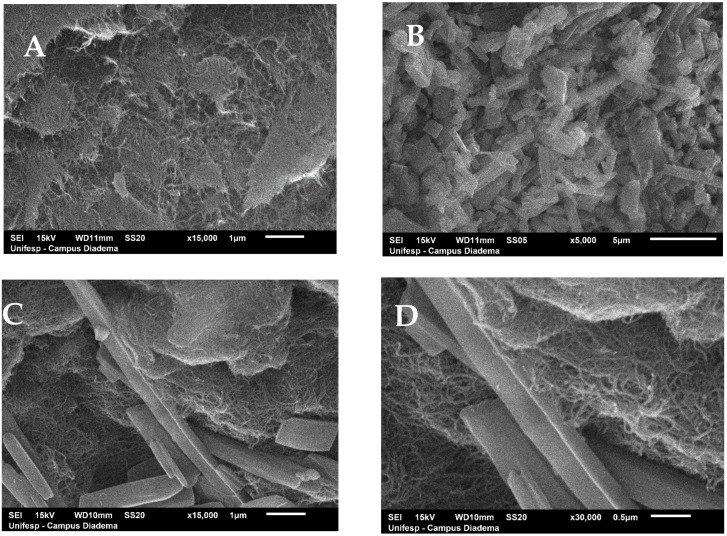
Scanning Electron Microscopy micrographs of CNT_HP_AA (**A**) (CNT after acid treatment), RUTIN (**B**), CNT_RUT_HP_AA (with resolution 1 µm) (**C**) and CNT_RUT_HP_AA (with resolution 0.5 µm) (**D**).

Images from SEM analysis CNT_HP_AA, RUTIN and CNT_RUT_HP_AA are provided in Figure 2. The external morphology of the material was completed by SEM. Figure 2A depicts the tubular shape arranged in a three-dimensional structure. Figure 2B reveals the crystalline structure of pure drugs as small rod-like crystals [28]. Figure 2C,D shows the CNTs fenced in the rutin.

Thermal analysis has a close relationship with the quality, safety, and efficacy of nanopharmaceuticals. They have been widely used as a tool for the evaluation of preformulations, from the identification of polymorphisms, purity, compatibility, and definition of stability of drugs and pharmaceutical formulation. These uses make thermal analysis a fundamental tool for the characterization of pharmaceuticals.

Differential scanning calorimetry (DSC) is a technique that measures the difference in energy supplied to a given substance and material as a function of temperature and/or time, while the sample is subjected to controlled temperature programming. The thermal events that generate changes in DSC curves can basically be first and second order transitions. First-order transitions show enthalpy variations—endothermic or exothermic—and give rise to peak formation.

**Figure 3:**
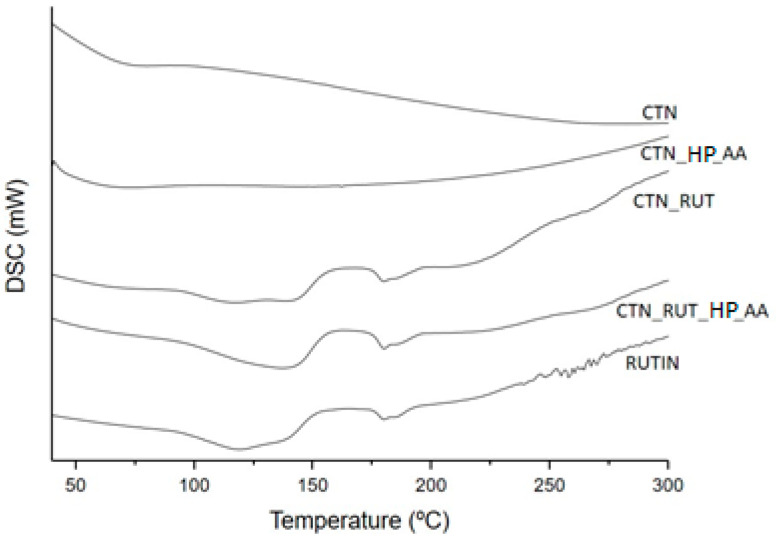
Differential scanning calorimetry (DSC) curves of CNT (carbon nanotube), CNT_HP_AA (CNT after acid treatment), CNT_RUT, CNT_RUT_HP_AA, and RUTIN.

The obtained DSC curves are shown in Figure 3. The DSC profile of CNT_HP_AA, and CNT samples did not show any enthalpic event up to 300 °C.

The DSC curves of rutin show three consecutive stages of mass loss, the first endothermic started at 56.05 °C and completed at 150.39 °C, attributed to a water loss, and the second started at 175.68 °C and finished at 191.85 °C, attributed to the melting of rutin (Table 2). A similar result was observed by [28]. However, these peaks were displaced and less perceived in the CNT_RUT and CNT_RUT_HP_AA curves.

**Figure 4:**
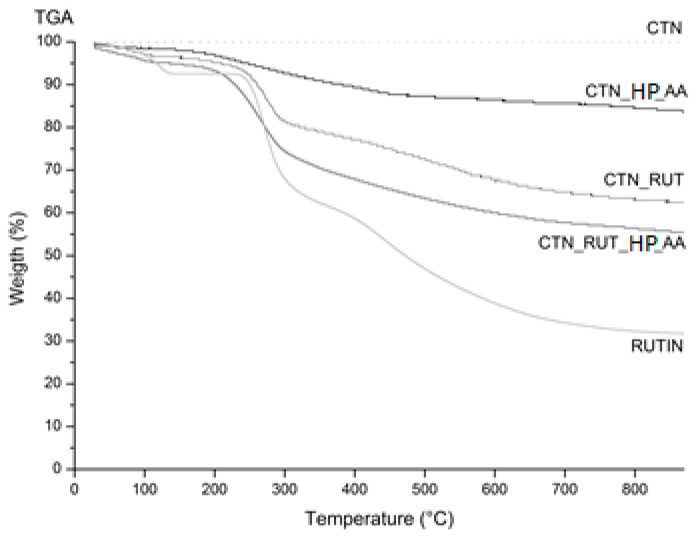
Thermogravimetric and DTA analyses of CNT, CNT_HP_AA, CNT_RUT, CNT_RUT_HP_AA, and RUTIN.

The thermogravimetric curves of the CNT functionalized with rutin are depicted in Figure 4, and mass loss is summarized in Table 3. The TG isothermal profile of rutin presented two decomposition stages as the first stage occurred at ~100 °C due to loss of superficial moisture. The other stage refers to the melting phase, which occurred between 240 and 330 °C. At a higher temperature, the weight loss continued due to the degradation, while in the CNT_RUT_HP_AA, the elimination of water and degradation occurred at the same temperature, but the weight loss was reduced compared to rutin. In CNTs, no alteration was observed as expected. The TGA data agreed well with DSC.

**Figure 5:**
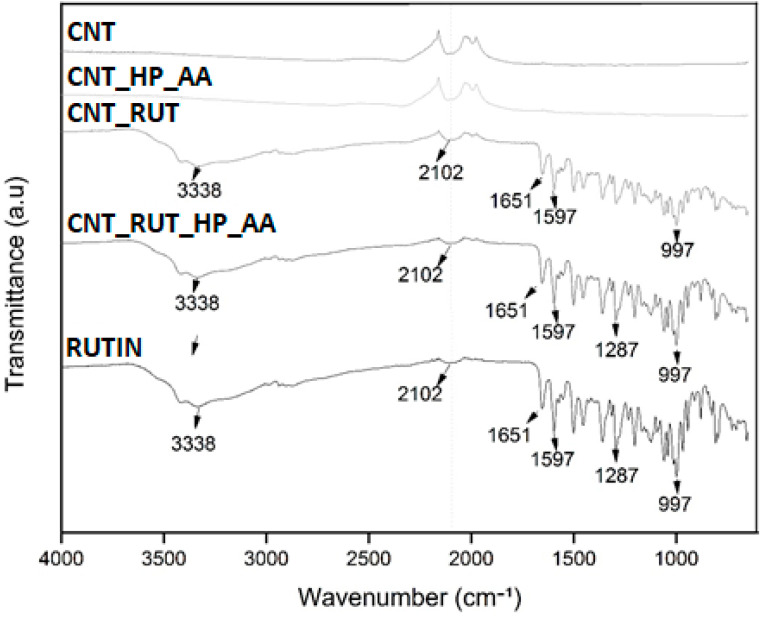
Fourier transform infrared spectroscopy spectra of CNT, CNT_HP_AA, CNT_RUT, CNT_RUT_HP_AA, and RUTIN.

The interaction of functional groups and formation of end-products were analysed by FTIR. The results obtained from FTIR analyses corroborate the results from molecular docking, confirming the involvement of intermolecular by hydrogen bonds. The spectra of the CNT samples represented by Figure 5 showed peaks between 2500 and 1800 cm^−1^ corresponding to the elongation of the nitrile group, resulting from the use of nitric acid used in the functionalization of the CNTs.

The bands widened between 3500 and 3252 cm^−1^ referring to the striation band of the group (OH), mainly O–H stretching bands at 3338 cm^−1^, C–H vibrations near 2974 cm^−1^ and 997 cm^−1^, and C–O stretches corresponding at 1300 to 1000 cm^−1^ attributed to phenolic and carboxylic groups of rutin [29]. Stretch bands suggestive of connections (C=O), a marked ester characteristic, appeared between 1750 and 1735 cm^−1^ [30].

**Figure 6:**
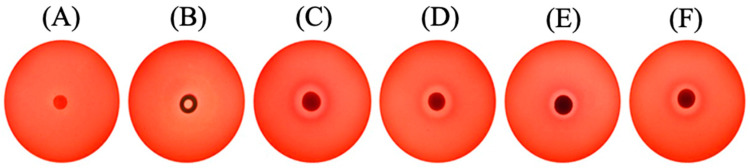
Results of the cytotoxicity analysis using diffusion disk in agar and 3T3 cells. (**A**) Negative control (DMEM medium); (**B**) Positive control (DMSO); (**C**) Rutin; (**D**) CNT, (**E**) CNT_RUT_HP_AA 100 µg and (**F**) CNT_RUT_HP_AA 1000 µg.

The cytotoxicity potential of rutin, CNT, CNT_RUT_HP_AA via the agar disk diffusion methodology using the cell lineage 3T3 is shown in Figure 6. The results obtained showed that there was mild cytotoxicity in all samples, presented by the clearer halo around all the material samples.

When compared to the literature, this preliminary study showed an indication of adjustments in the concentration of both CNT and rutin. Ponti et al. (2013) [31] investigated the toxicological effects of multiwall CNTs in 3T3 fibroblast cells confirming a dose-dependent relationship upon exposure. In addition, CNTs formed aggregates of filamentous structures on top of the cells. Sohaebuddin et al. (2010) [32] suggested that the cytotoxicity of CNTs is dependent on the target cell, size, and composition of the nanoparticles.

## 4. Conclusions

Our study demonstrates the feasibility of developing CNT anchored with rutin. The physicochemical characterization suggests hydrogen bonding interaction between CNTs and rutin, which contributes to the stabilization of this flavonoid. Cytotoxicity of surface-tailored CNTs was found to be dose-dependent, suggesting the need to adjust the ratio between CNTs and rutin to ensure the reduction of cells’ oxidative stress. Cell viability studies using other distinct cell lines are recommended together with additional physicochemical characterization as nuclear magnetic resonance to further confirm the suitability of rutin-coated CNTs as an alternative drug delivery system.

## Figures and Tables

**Table 1:** Docking affinity energy, type of interaction and geometry distance (Å) for CNT_HP_AA_RUTIN.

Affinity (kcal/mol)	Type of Interaction	From	To	Distance (Å)	Angle DHA	Angle HAY
−8.5	Hydrogen Bond	CNT_HP_AA	Rutin	2.83	146.8	105.2
				2.39	113.6	96.0
				2.42	99.2	99.1
				2.80	125.4	105.4
				2.70	157.8	108.9
				2.76	101.2	108.6
		Rutin	CNT_HP_AA	2.22	158.9	93.5
				2.46	126.7	99.6
				1.96	105.3	111.6
		CNT_HP_AA	Rutin	2.57	146.6	99.5
				3.00	114.3	97.3

**Table 2:** Thermal properties of CNT, CNT_HP_AA, CNT_RUT, CNT_RUT_HP_AA, and RUTIN.

Sample	Events	T_onset_ (°C)	T_endset_ (°C)	∆H (J)
CNT	1	35.80	475.90	−4.12
CNT_HP_AA	1	24.69	93.51	−0.22
	2	138.59	453.18	−1.41
CNT_RUT	1	25.58	144.69	−0.12
	2	145.80	177.68	0.05
	3	177.70	194.79	−0.01
CNT_RUT_HP_AA	1	32.39	154.77	−0.34
	2	174.96	187.49	−0.03
RUTIN	1	56.05	150.39	−0.30
	2	175.68	191.85	−0.01

**Table 3:** Mass loss obtained by thermogravimetric analysis of CNT, CNT_HP_AA, CNT_RUT, CNT_RUT_HP_AA, and RUTIN.

Sample	∆_m1_ (%) 30–170 °C	∆_m2_ (%) 170–240 °C	∆_m3_ (%) 240–340 °C	∆_m4_ (%) 340–800 °C
CNT	0	0	0	0
CNT_HP_AA	0.78	2.08	4.63	8.55
CNT_RUT	0.58	2.92	17.97	18
CNT_RUT_HP_AA	1.32	5.48	11.79	28.65
RUTIN	6.58	7.52	7.73	36.63

## Data Availability

Data are available from authors upon request.
